# Expired Ibrutinib as a Sustainable Corrosion Inhibitor for P110 Carbon Steel in Hydrochloric Acid: Integrated Experimental, Electrochemical, and Multiscale Computational Insights

**DOI:** 10.3390/molecules31173013

**Published:** 2026-08-28

**Authors:** Halima A. Alrafai, Ismat H. Ali, Mahmoud A. Bedair

**Affiliations:** 1 Chemistry Department, College of Science, King Khalid University, P.O. Box 9004, Abha 61341, Saudi Arabia; halrafai@kku.edu.sa; 2 Department of Chemistry, College of Science, University of Bisha, P.O. Box 511, Bisha 61922, Saudi Arabia; mbedair@ub.edu.sa

**Keywords:** ibrutinib, P110 carbon steel, PDP, EIS, DFT calculations, molecular simulations

## Abstract

The reuse of expired pharmaceuticals as corrosion inhibitors offers a sustainable strategy for reducing pharmaceutical waste while providing environmentally friendly alternatives to conventional inhibitors. In this work, the corrosion inhibition performance of expired ibrutinib (EIB) for P110 carbon steel in 1.0 M HCl was investigated using electrochemical techniques, mass loss measurements, surface characterization, and computational approaches. Electrochemical impedance spectroscopy (EIS) revealed a progressive increase in charge-transfer resistance with increasing inhibitor concentration, while potentiodynamic polarization (PDP) measurements demonstrated that EIB acts as a mixed-type inhibitor with a predominant anodic effect. At 1000 mg L^−1^, inhibition efficiencies of 88.7%, 96.8%, and 93.3% were obtained from EIS, PDP, and mass loss measurements, respectively. SEM analysis confirmed the formation of a compact and homogeneous protective film on the steel surface, significantly reducing corrosion damage and surface roughness. Density functional theory (DFT), Natural Bond Orbital (NBO), Monte Carlo (MC), and molecular dynamics (MD) simulations demonstrated strong adsorption of EIB on the Fe(110) surface through nitrogen- and oxygen-containing active centers, while radial distribution function analysis confirmed the contribution of chemisorption. The excellent agreement between the experimental and theoretical results demonstrates that expired ibrutinib is an efficient and sustainable corrosion inhibitor for P110 carbon steel in acidic environments and represents a promising approach for the valorization of expired pharmaceutical products.

## 1. Introduction

Carbon steel (CS) is widely used in industrial applications due to its cost-effectiveness, mechanical properties, and availability. However, its susceptibility to corrosion in acidic environments, such as hydrochloric acid (HCl), presents a significant challenge, particularly in industries like oil and gas, chemical processing, and metal cleaning. In acidic media, CS undergoes rapid dissolution due to the aggressive action of hydrogen ions, leading to material degradation, reduced structural integrity, and increased maintenance costs. To mitigate this issue, the use of corrosion inhibitors is a well-established practice. These substances slow down or prevent metal deterioration by forming protective layers on the surface. However, concerns over the environmental impact and toxicity of conventional inhibitors have driven research into sustainable and biodegradable alternatives [1,2,3].

Expired pharmaceuticals often have complex molecular structures, especially those containing heteroatoms like nitrogen and oxygen, multiple bonds, and aromatic rings that make them attractive as eco-friendly corrosion inhibitors. These structural elements allow the compounds to bond with iron ions on metal surfaces, forming stable coordination complexes that act as a protective layer, helping to shield the metal from corrosion in harsh environments. Additionally, the electron-donating properties of these functional groups enhance adsorption efficiency, further improving inhibition performance. Unlike traditional inhibitors, the biodegradability of these pharmaceuticals offers an eco-friendly alternative, reducing their ecological footprint and providing an innovative way to repurpose pharmaceutical waste [4,5].

Improperly discarding expired medications can lead to serious environmental problems, such as polluting water sources, degrading soil quality, and endangering wildlife. In addition, it may promote the development of antibiotic-resistant bacteria and other dangerous microorganisms, posing risks to both ecosystems and public health. Many healthcare facilities and industries lack adequate waste management systems to handle expired medications effectively. Repurposing such drugs as corrosion inhibitors not only offers an environmentally responsible solution but also addresses the pressing need for sustainable alternatives in industrial corrosion protection [6].

This study investigates the use of an expired pharmaceutical product as an effective and eco-friendly corrosion inhibitor for carbon steel in a hydrochloric acid medium. The research highlights the dual benefits of utilizing pharmaceutical waste to mitigate corrosion while addressing environmental concerns.

Recent advancements have highlighted the potential of expired pharmaceutical products as sustainable anti-corrosion agents for metals in acidic environments. These studies leverage the unique chemical structures of pharmaceuticals, which often include heteroatoms (e.g., N, O) and aromatic systems capable of forming strong adsorptive layers on metal surfaces, offering an eco-friendly alternative to traditional inhibitors [7,8].

It has been reported that [9] the use of expired favipiravir for aluminum–silicon alloy corrosion inhibition in 1 M HCl achieves 96% efficiency at 100 ppm. This performance was attributed to the drug’s ability to adsorb onto the alloy’s surface, forming a protective film. Similarly, it has been mentioned that [10] midazolam is a green inhibitor for copper in 0.1 M nitric acid, achieving 93% efficiency. The inhibitor followed the Langmuir adsorption isotherm, highlighting its strong interaction with the metal surface. Alnumani et al. [11] explored an ethanolic extract of Foeniculum vulgare and Pimpinella anisum that extracts steel in 0.1 M HCl, reporting 99% inhibition efficiency due to its mixed-type inhibition behavior.

Other studies have reinforced the potential of pharmaceuticals as corrosion inhibitors. Bhawsar and his co-workers [12] reported the corrosion inhibition potential of expired ivermectin on mild steel. Similarly, it has been reported that [13] expired gababentin was used as a corrosion inhibitor. The results indicate that Gabapentin is a highly effective corrosion inhibitor, achieving an inhibition efficiency of 90.3% at a concentration of 400 ppm and a temperature of 303 K. Its adsorption onto the metal surface involves both physical and chemical interactions, as supported by the Langmuir adsorption isotherm model. Electrochemical studies further reveal that Gabapentin functions as a mixed-type inhibitor, influencing both anodic and cathodic corrosion processes. Furthermore, Sheetal et al. [14] explored the use of Pyridoxine hydrochloride as an inhibitor for mild steel in HCl, achieving efficiency above 85% due to the drug’s nitrogen- and oxygen-rich functional groups.

Despite these encouraging findings, key limitations persist. Many studies focus on specific drugs without exploring broader pharmaceutical categories or their synergistic effects. Additionally, limited attention has been given to the biodegradability of these inhibitors and the environmental impact of their degradation products in corrosive environments. Most research also lacks molecular-level analysis, leaving gaps in understanding the structure–performance relationship. Long-term stability and scalability of these inhibitors in industrial conditions remain largely unaddressed [15].

**Scheme 1:**
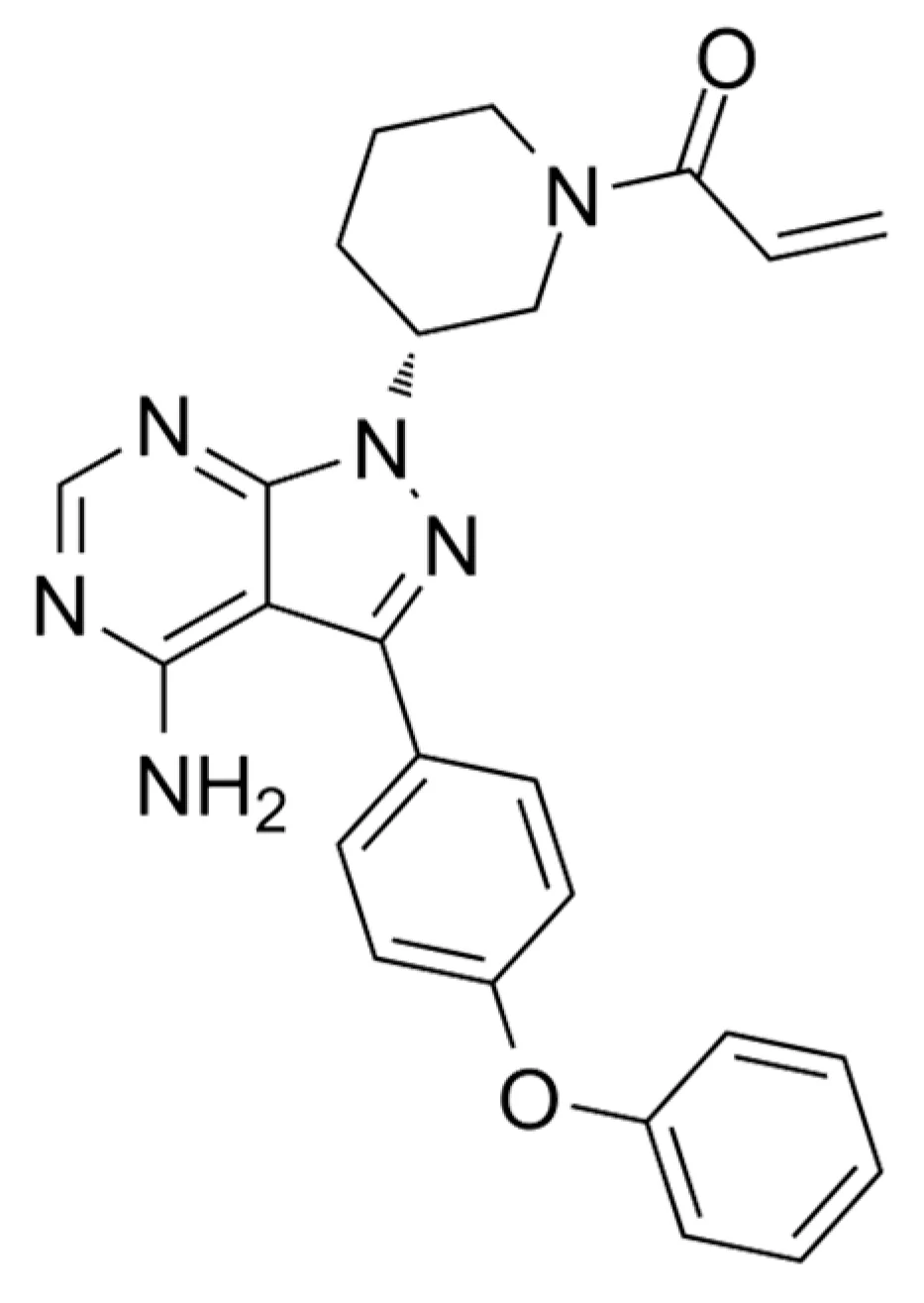
Chemical structure of ibrutinib.

Current research has yet to explore the application of expired pharmaceutical products as effective corrosion inhibitors for carbon steel in highly acidic environments, such as 1 M HCl, with a focus on detailed mechanistic studies. Moreover, the relationship between the inhibitor’s chemical structure and its inhibition performance is not fully understood. This study aims to investigate expired ibrutinib (Scheme 1) as a potential corrosion inhibitor for P110 carbon steel in an acidic medium through electrochemical, gravimetric, surface characterization, and computational approaches, while providing molecular-level insights into its inhibition behavior.

This study aimed to investigate the potential of expired ibrutinib as an eco-friendly and effective alternative to conventional corrosion inhibitors. The investigation involved electrochemical tests and theoretical studies to evaluate the effectiveness of expired ibrutinib as an efficient inhibitor in an aggressive corrosive environment. 

## 2. Results

### 2.1. FTIR Spectroscopy

**Figure 1:**
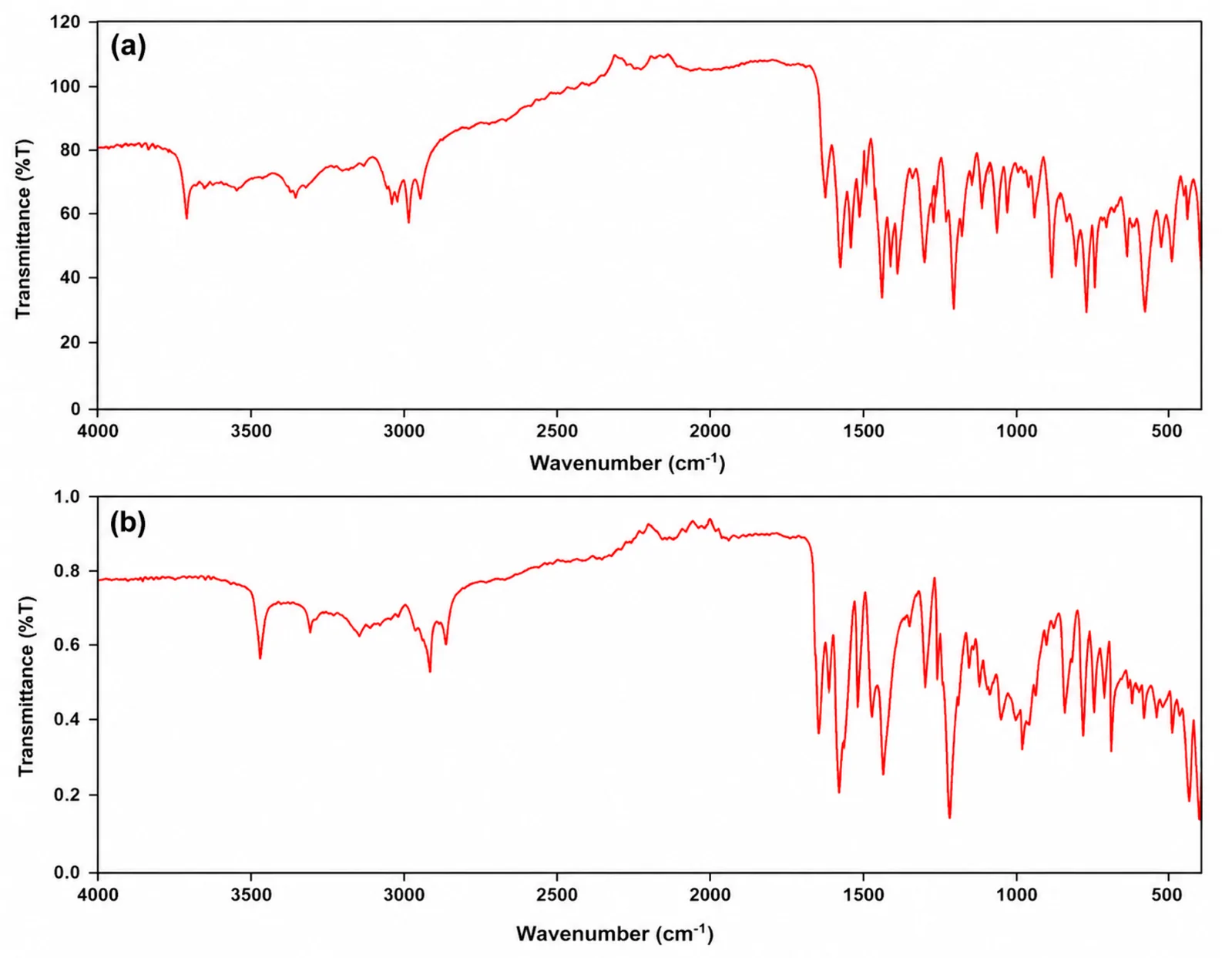
Comparison of FTIR spectra of (**a**) pure ibrutinib reported in the literature and (**b**) expired ibrutinib used in the present study.

Figure 1 compares the FTIR spectrum of the expired ibrutinib (EIB) used in the present study with the reference spectrum of pure ibrutinib. Overall, the two spectra exhibit comparable spectral features over the investigated wavenumber range, particularly in the regions associated with the main functional groups of the ibrutinib molecule. The characteristic absorptions in the high-wavenumber region can be associated with N–H and C–H stretching vibrations, while the bands occurring at lower wavenumbers are attributable to vibrations involving the conjugated and heteroatom-containing functionalities of the molecule. In particular, the absorption features in the approximately 1700–1500 cm^−1^ region can be related to C=O/C=N and aromatic C=C vibrations, whereas the complex fingerprint region below approximately 1500 cm^−1^ contains contributions from C–N, C–O, and aromatic/heterocyclic skeletal vibrations. The persistence of these characteristic spectral regions in the expired sample, together with the overall similarity to the reference spectrum of pure ibrutinib, indicates that the principal molecular framework and major functional groups of ibrutinib remain detectable after expiration.

This observation is particularly relevant to the corrosion-inhibition behavior of EIB because the retained heteroatom-containing groups and conjugated π-system provide potential interaction sites with the P110 steel/solution interface. Nitrogen- and oxygen-containing functionalities may participate in interactions with the metallic surface, while the aromatic and heterocyclic π-electron systems may further contribute to adsorption. Therefore, the preservation of these structural features is consistent with the experimentally observed corrosion-inhibition activity of the expired pharmaceutical. Nevertheless, the FTIR comparison should not be interpreted as evidence that the expired material is chemically identical to pure ibrutinib. Differences in relative band intensities and profiles can arise from several factors, including sample preparation, instrumental conditions, physical state, and possible changes occurring during storage.

More importantly, FTIR analysis alone cannot reliably identify or exclude minor degradation products that may have formed during or after the shelf life of the pharmaceutical. Consequently, although the spectral comparison supports the persistence of the major structural features of ibrutinib in the expired sample, the possible presence of degradation products cannot be ruled out in the absence of a more selective compositional technique such as LC–MS. Such species, if present, may also contain heteroatoms and/or π-electron-rich functionalities and could potentially contribute to the overall interaction of the expired pharmaceutical material with the steel surface. Accordingly, the inhibition mechanism observed in the present work is more appropriately attributed to the expired ibrutinib material as experimentally tested, rather than exclusively to chemically unchanged ibrutinib molecules. The present FTIR results therefore provide evidence for retention of the principal functional groups relevant to interfacial interactions, while the precise contribution of any minor degradation products to the corrosion-inhibition process remains to be established by future compositional studies [16,17].

### 2.2. Electrochemical Measurements

#### 2.2.1. OCP Test

**Figure 2:**
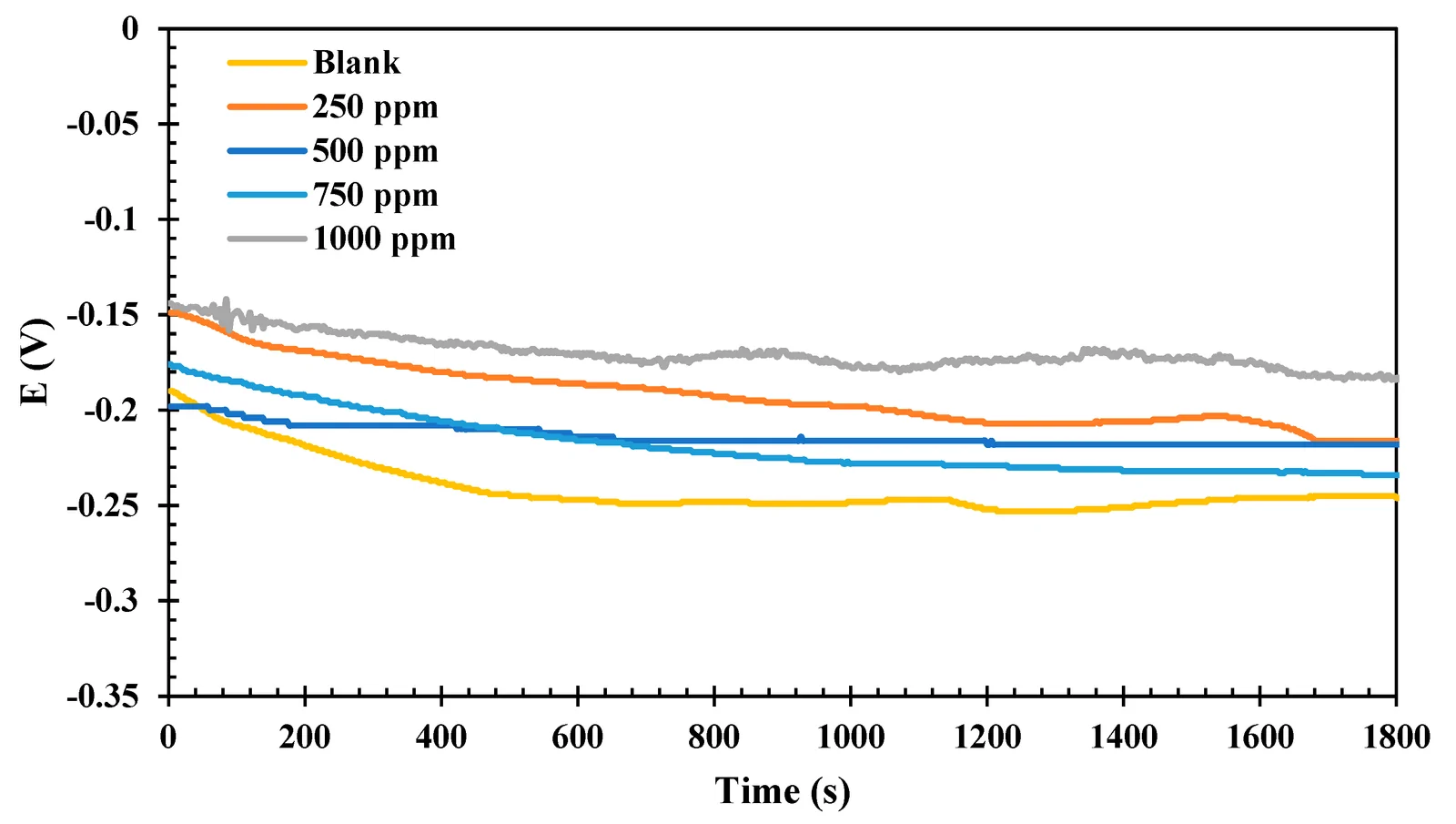
OCP diagrams of CS P110 immersed in 1.0 M HCl solutions with various EIB concentrations.

To assess the electrochemical stability of the samples, the open-circuit potential (OCP) of CS coupons in acidic media was measured with varying inhibitor dosages, and the resulting curves are shown in Figure 2. Over time, the OCP values of the CS coupon in the blank solution shifted to more negative potentials. This behavior can be attributed to the breakdown of the pre-existing oxide film on the steel surface due to the aggressive acidic environment. As this protective layer deteriorates, active sites such as defects, grain boundaries, or surface imperfections become exposed, making them more prone to metal dissolution. Consequently, the electrode displayed increasingly active behavior with longer immersion time.

In contrast, samples containing the inhibitor exhibited a less pronounced decrease in OCP values, suggesting the formation of a protective organic film on the electrode surface. The fluctuations observed in the OCP curves of EIB-containing samples result from dynamic processes at the solid/solution interface. Localized aggressive species may temporarily weaken the inhibitor film, causing intermittent breakdowns and fluctuations in OCP during immersion. When comparing the OCP values of samples with and without the inhibitor, it is evident that the inhibitor reduces the shift toward negative potentials, especially with longer soaking times, indicating enhanced protection of the steel surface [18].

#### 2.2.2. EIS Measurements

**Figure 3:**
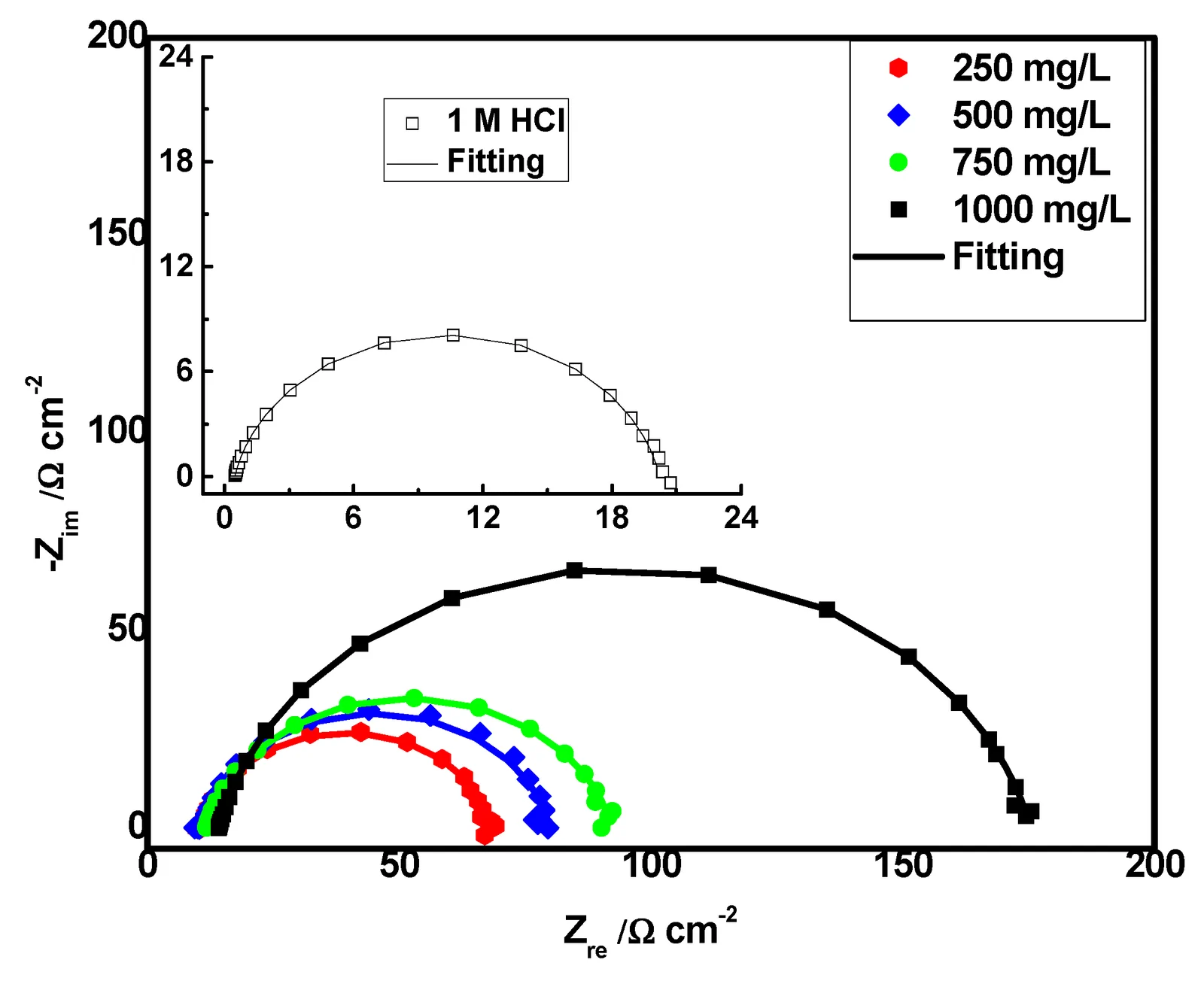
Nyquist impedance plots of P110 carbon steel in 1.0 M HCl solution without and with different concentrations of EIB.

Figure 3 presents the Nyquist plots of P110 carbon steel in 1.0 M HCl solution in the absence and presence of different concentrations of EIB, while the corresponding electrochemical parameters obtained from equivalent circuit fitting are summarized in Table 1. All impedance spectra exhibit a single depressed capacitive semicircle, indicating that the corrosion process is predominantly governed by charge transfer at the steel/solution interface. The diameter of the capacitive loop increases continuously with increasing EIB concentration, demonstrating a progressive improvement in corrosion resistance due to adsorption of the inhibitor on the steel surface. This behavior is confirmed by the increase in the charge-transfer resistance (R_ct_), which is calculated using Equation (1), from 18.3 Ω cm^−2^ for the blank solution to 57.8, 75.1, 99.2, and 161.3 Ω cm^−2^ in the presence of 250, 500, 750, and 1000 mg L^−1^ EIB, respectively, as follows:

(1) $$IE\%=\;\frac{R_{ct}^{i}-R_{ct}^{o}}{R_{ct}^{i}}\;\times 100$$

where $R_{ct}^{i}$ is the charge-transfer resistance in the presence of EIB, whereas $R_{ct}^{o}$ is the charge-transfer resistance without EIB.

Accordingly, the inhibition efficiency calculated from the EIS measurements increased from 67.5% to 88.7% with increasing inhibitor concentration, indicating that greater surface coverage was achieved at higher EIB concentrations. The constant phase element exponent (*n*) increased from 0.82 for the uninhibited solution to 0.94 at 1000 mg L^−1^, suggesting that the steel surface became progressively more homogeneous and exhibited a more ideal capacitive behavior as a result of the formation of a compact and uniform inhibitor film. Moreover, the double-layer capacitance (C_dl_), which is calculated using Equation (2), decreased markedly from 1124 to 382 µF cm^−2^ after the addition of EIB [19,20].

This reduction in C_dl_ is attributed to the adsorption of EIB molecules on the steel surface, which replaces water molecules at the interface, lowers the local dielectric constant, and/or increases the thickness of the electrical double layer. Although slight fluctuations in C_dl_ were observed at intermediate concentrations, the overall decrease compared with the blank confirms the formation of a protective adsorbed layer. The EIS results therefore demonstrate that EIB effectively inhibits the corrosion of P110 carbon steel by forming a stable barrier film that significantly impedes the charge-transfer process. These findings are in good agreement with the potentiodynamic polarization and gravimetric measurements, which likewise showed that the inhibition performance improves with increasing inhibitor concentration:

(2) $$C_{dl}={{(Y}_{o,\;dl})}^{(1/n)}\;\times \;{\left[\frac{R_{ct}\;\times {\;R}_{s}}{R_{ct}+R_{s}}\right]}^{\left((1-n\right)/n)}$$

**Figure 4:**
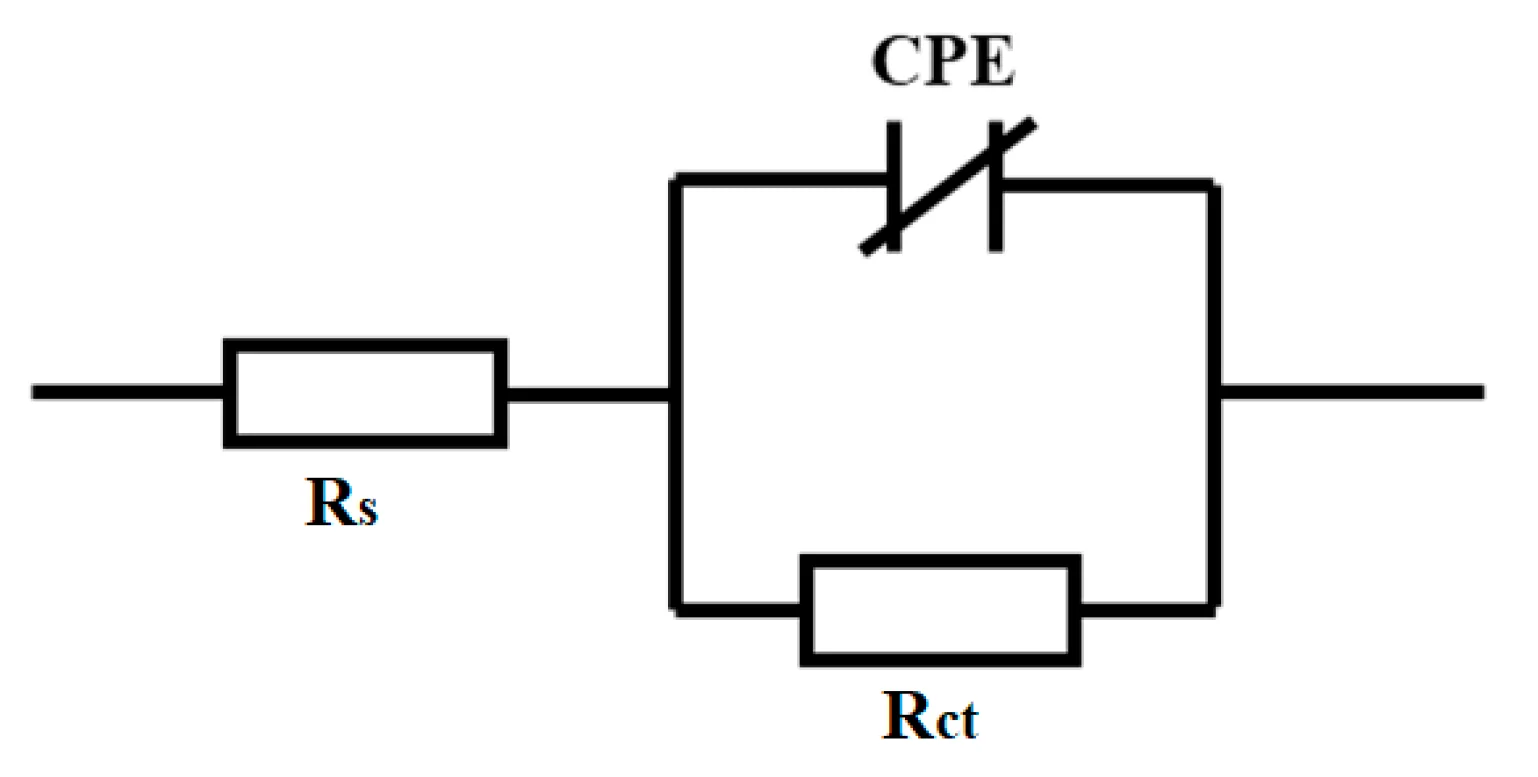
Equivalent electrical circuit used to fit the electrochemical impedance spectroscopy (EIS) data of P110 carbon steel in 1.0 M HCl solution.

The EIS spectra were fitted using the equivalent circuit shown in Figure 4, consisting of the solution resistance (R_s_), the charge-transfer resistance (R_ct_), and a constant phase element (CPE). The CPE was used instead of an ideal capacitor to account for the non-ideal capacitive behavior arising from surface roughness, heterogeneity, and non-uniform adsorption of the inhibitor on the steel surface [20].

#### 2.2.3. PDP Analysis

**Figure 5:**
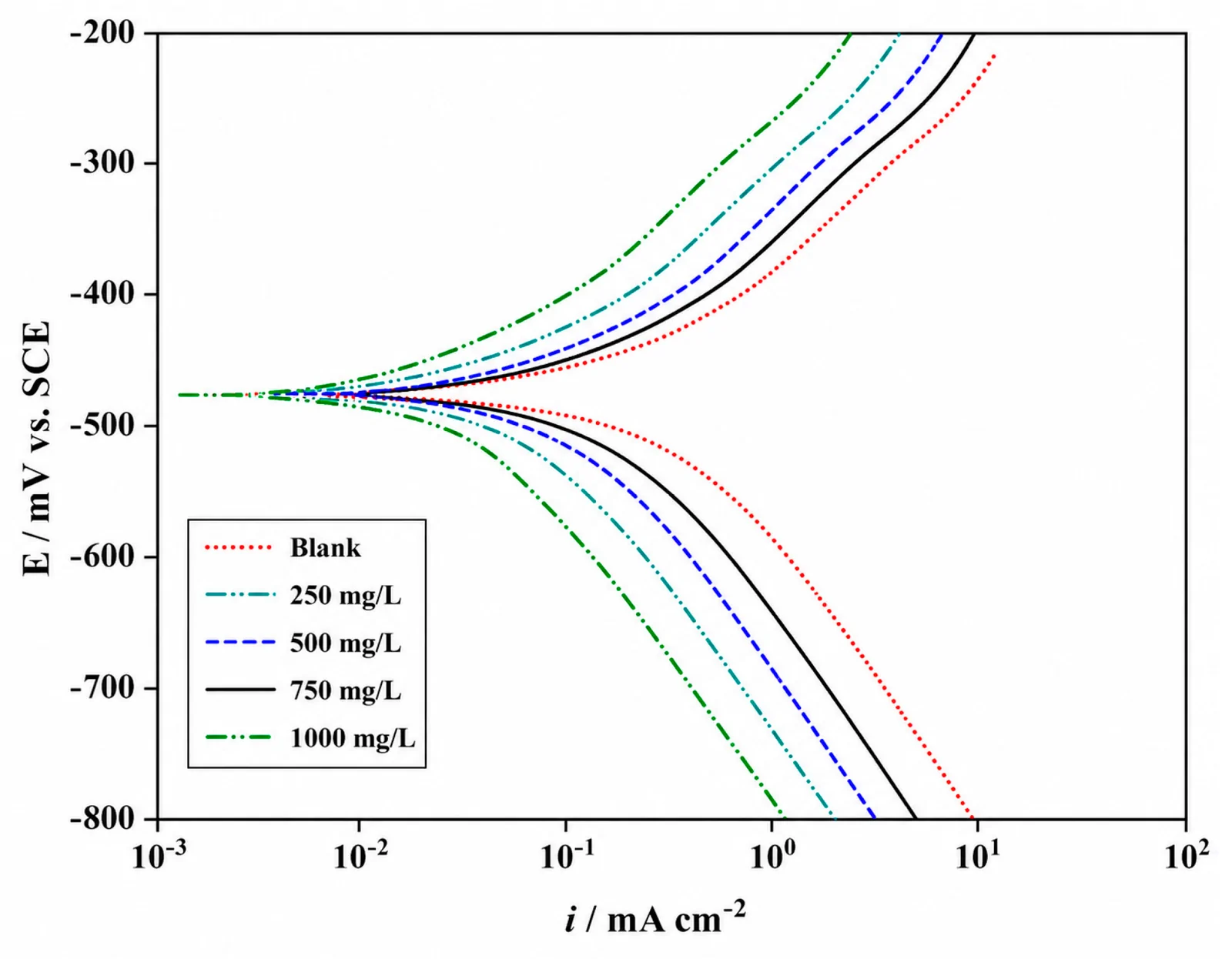
Potentiodynamic polarization curves of P110 carbon steel immersed in 1.0 M HCl in the absence and presence of different concentrations of EIB.

Figure 5 presents the potentiodynamic polarization curves of P110 carbon steel in 1.0 M HCl solution in the absence and presence of different concentrations of EIB, while the corresponding electrochemical parameters are summarized in Table 2. The addition of EIB markedly reduced both the anodic and cathodic current densities, indicating that the inhibitor effectively suppresses the electrochemical reactions responsible for steel corrosion. As the inhibitor concentration increased from 250 to 1000 mg L^−1^, the corrosion current density (i_corr_) decreased significantly from 1.954 μA cm^−2^ for the blank solution to 0.609, 0.536, 0.297, and 0.062 μA cm^−2^, respectively, demonstrating a progressive reduction in the corrosion rate. Consequently, the inhibition efficiency increased from 68.9% at 250 mg L^−1^ to 96.8% at 1000 mg L^−1^, confirming the excellent corrosion inhibition performance of EIB. Simultaneously, the polarization resistance (R_p_) increased from 15.5 kΩ cm^2^ for the uninhibited solution to 491.1 kΩ cm^2^ at the highest inhibitor concentration, further confirming the formation of a highly protective surface film that effectively hinders the charge-transfer process. The anodic and cathodic Tafel slopes changed only slightly in the presence of EIB, suggesting that the inhibitor does not alter the fundamental corrosion mechanism but rather reduces the reaction rates by blocking active sites on the steel surface. The Tafel slopes showed only small variations with EIB concentration, with β_a_ ranging from 0.115 to 0.117 V dec^−1^ and ∣β_c_∣ from 0.174 to 0.177 V dec^−1^. The nearly unchanged slopes suggest that EIB suppresses both anodic iron dissolution and cathodic hydrogen evolution mainly by reducing their reaction rates rather than altering their fundamental mechanisms. The corrosion potentials remained close to −0.48 V vs. SCE for all inhibitor concentrations, indicating no substantial displacement of E_corr_. The progressive decrease in corrosion current density together with the increase in polarization resistance demonstrates that adsorption of EIB molecules forms a compact and stable adsorbed layer on the P110 steel surface, effectively isolating the metal from the aggressive hydrochloric acid environment. These results are in good agreement with the EIS and gravimetric measurements, which likewise show that the inhibition efficiency improves with increasing inhibitor concentration owing to the adsorption of EIB on the steel surface [21].

### 2.3. Mass Loss Test

The gravimetric measurements confirmed that EIB is an effective corrosion inhibitor for P110 carbon steel in 1.0 M HCl solution, with its inhibition performance improving progressively as the inhibitor concentration increased. As presented in Table 3, the corrosion rate (expressed as mass loss) decreased significantly in the presence of EIB. Accordingly, the inhibition efficiency increased, indicating that the inhibition performance is strongly concentration-dependent. The marked decrease in corrosion rate with increasing inhibitor concentration suggests that a larger number of EIB molecules adsorb onto the steel surface, forming a compact and protective adsorbed film that effectively suppresses the dissolution of P110 steel in the acidic medium. The highest inhibition efficiency demonstrates the excellent protective performance of EIB. Furthermore, the inhibition efficiencies obtained from the mass loss measurements are in good agreement with those determined by EIS and PDP measurements, confirming the reliability and consistency of the proposed corrosion inhibition mechanism across different experimental techniques [21,22]. The gravimetric measurements clearly demonstrated the concentration-dependent inhibition behavior of EIB over the investigated concentration range. At relatively low concentrations of 50 and 100 mg L^−1^, inhibition efficiencies of 42.1% and 53.9%, respectively, were obtained. Increasing the EIB concentration to 250, 500, 750, and 1000 mg L^−1^ progressively enhanced the inhibition efficiency to 63.9, 74.2, 82.4, and 93.3%, respectively. This consistent trend indicates that increasing the EIB concentration promotes greater adsorption at the steel/solution interface, leading to more effective suppression of the corrosion process.

### 2.4. Temperature Effect

The effect of temperature on the corrosion behavior of P110 carbon steel in 1.0 M HCl was investigated in the absence and presence of 1000 mg L^−1^ EIB over the temperature range of 298–328 K. As summarized in Table 4, the corrosion rate increased with increasing temperature for both the blank and inhibited solutions, confirming that the dissolution of P110 steel is a thermally activated process.

**Figure 6:**
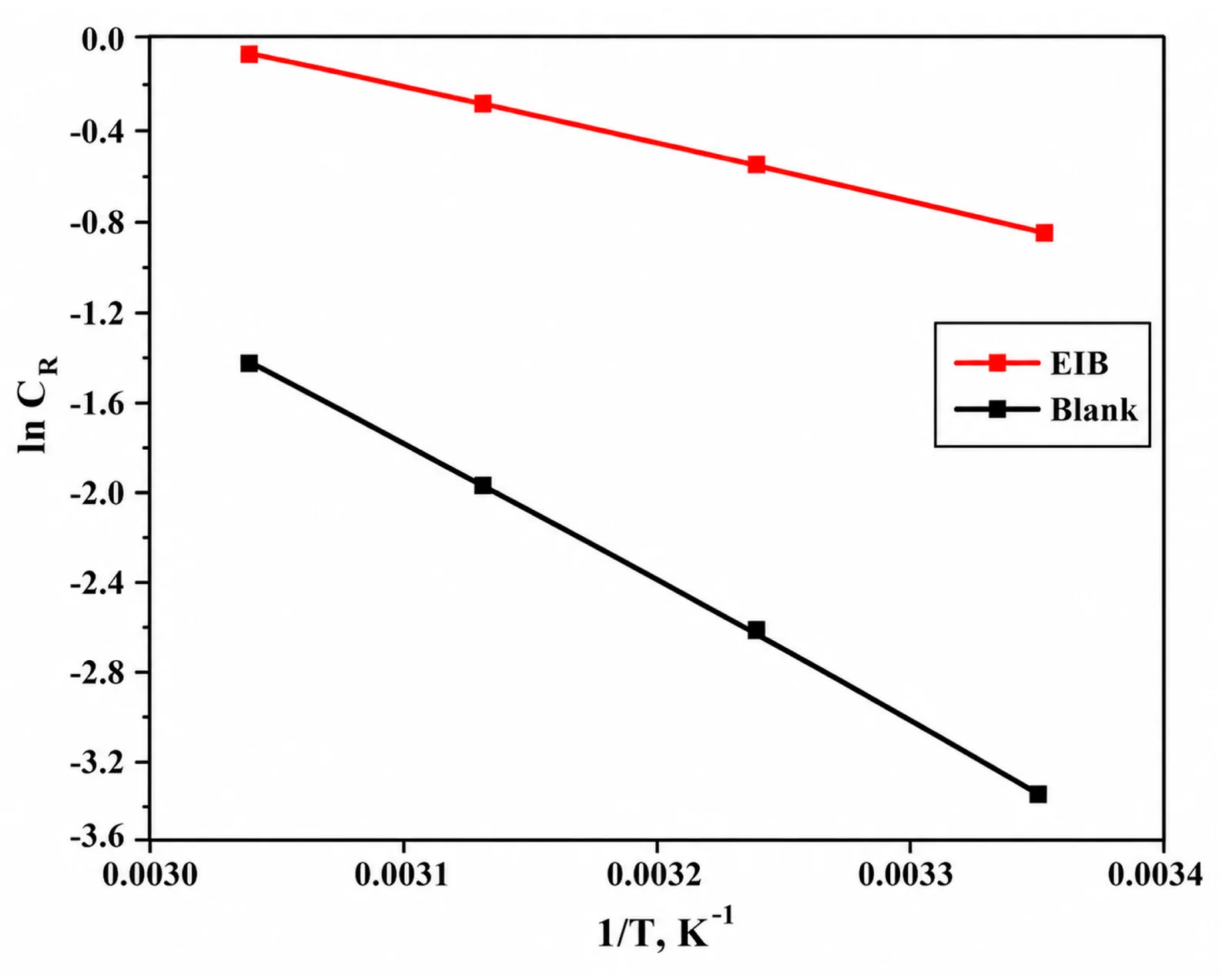
Arrhenius plots for P110 carbon steel in 1.0 M HCl without and with 1000 mg L^−1^ EIB.

In the uninhibited solution, the corrosion rate increased from 0.4922 to 0.9770 mg cm^−2^ h^−1^, whereas in the presence of EIB it increased only from 0.0329 to 0.1725 mg cm^−2^ h^−1^, indicating that the inhibitor retained excellent protection throughout the investigated temperature range. The Arrhenius plots (Figure 6) exhibited excellent linearity, demonstrating that the corrosion process follows the Arrhenius equation. The apparent activation energy (E_a_) decreased from 44.35 kJ mol^−1^ for the blank solution to 18.35 kJ mol^−1^ in the presence of EIB. Although a lower apparent E_a_ in the inhibited system indicates that the kinetic characteristics of the corrosion process are modified by inhibitor adsorption, this parameter alone is insufficient to establish the detailed reaction pathway. Moreover, the gradual increase in corrosion rate with temperature indicates that the protective effect of EIB becomes weaker at elevated temperatures, most likely because of partial desorption or reduced stability of the adsorbed inhibitor layer. Therefore, no conclusion regarding the thermal stability or exact nature of the surface film is drawn solely from the activation-energy data. Furthermore, the standard free energy of adsorption (${∆G}_{ads}^{o}$) was calculated to be −29.1 kJ mol^−1^ at 298 K, confirming that the adsorption process is spontaneous. The magnitude of ${∆G}_{ads}^{o}$ indicates that adsorption proceeds through a mixed mechanism involving both physical and chemical interactions. Electrostatic attraction between the protonated EIB molecules and the charged steel surface is expected to initiate adsorption, while subsequent interactions between the nitrogen- and oxygen-containing functional groups, the π-electron system of the aromatic rings, and the vacant d-orbitals of surface iron atoms further strengthen the adsorbed layer. This interpretation is fully consistent with the DFT calculations, which identified the heteroatoms and aromatic regions as the principal adsorption centers, and with the molecular dynamics simulations and RDF analysis, which demonstrated strong Fe–N and Fe–O interactions on the Fe(110) surface. Therefore, the combined thermodynamic, electrochemical, and computational results demonstrate that the outstanding inhibition performance of EIB originates from the formation of a compact and stable adsorbed film that effectively isolates the steel surface from the aggressive acidic environment [21].

### 2.5. SEM Micrograph

**Figure 7:**
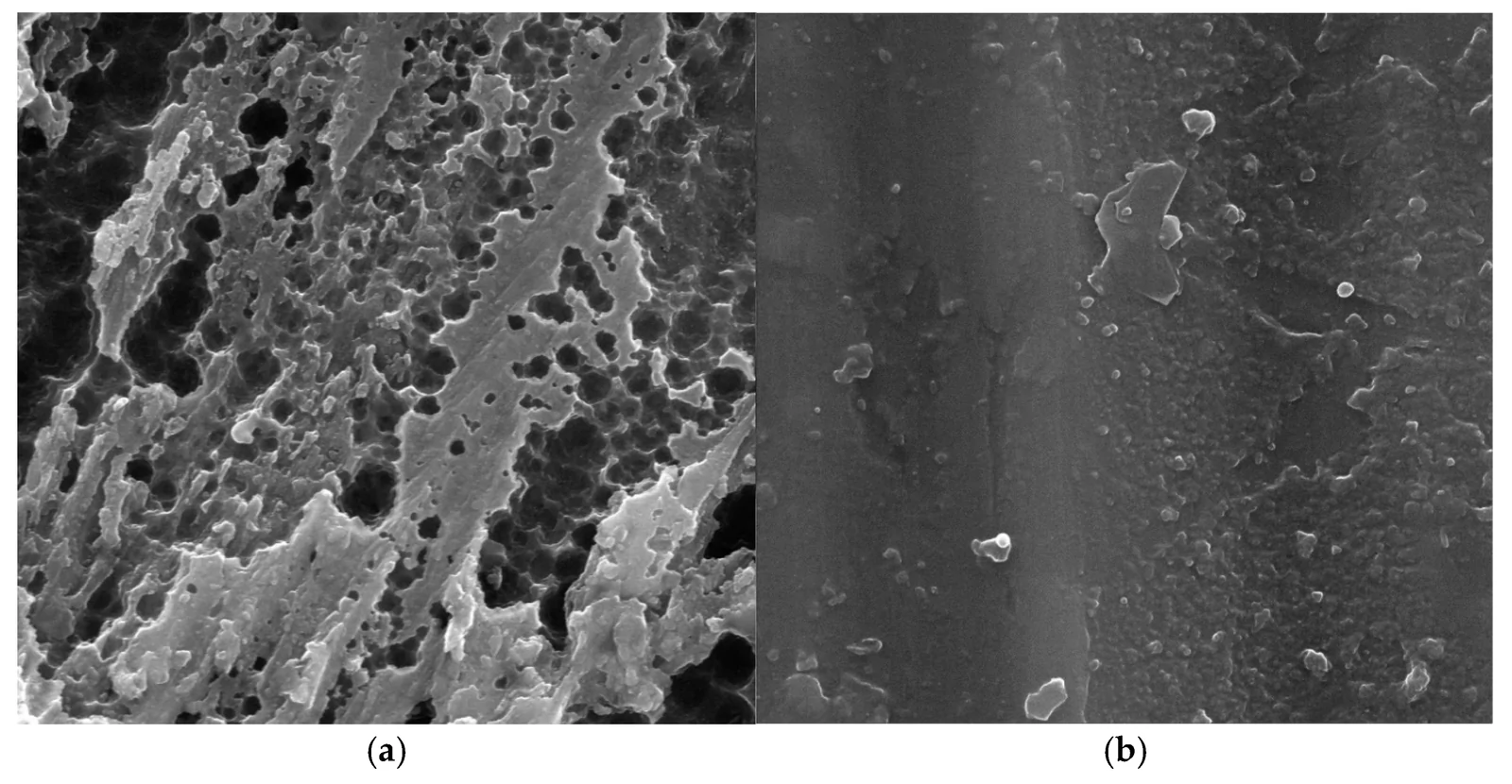
SEM micrographs of the immersed P110 CS in 1 M HCl without (**a**) and with (**b**) 1000 mg/L EIB.

The SEM micrographs shown in Figure 7 provide direct evidence for the protective effect of EIB on the corrosion behavior of P110 carbon steel in 1.0 M HCl solution. As shown in Figure 7a, the steel surface immersed in the uninhibited acid solution exhibits severe corrosion damage characterized by extensive pits, deep cavities, and a highly rough and porous morphology, indicating intense metal dissolution under the aggressive acidic conditions. In contrast, the specimen exposed to the solution containing 1000 mg L^−1^ EIB (Figure 7b) displays a much smoother and more uniform surface with significantly fewer corrosion defects. Although a few small corrosion products and isolated surface irregularities remain visible, the extensive pitting observed in the blank sample is largely suppressed.

The improved surface morphology indicates that adsorption of EIB molecules onto the steel surface modifies the interfacial electrochemical kinetics, thereby suppressing both anodic iron dissolution and cathodic hydrogen evolution. This behavior is consistent with an activation/activation-blocking inhibition mechanism rather than a purely physical blocking effect. These surface observations are in excellent agreement with the electrochemical (EIS and PDP) and gravimetric measurements, all of which demonstrate that EIB provides highly effective corrosion protection for P110 carbon steel through the formation of a stable adsorbed inhibitor layer [21].

### 2.6. Computational Results

#### 2.6.1. Prediction of the Major Microspecies of EIB

**Figure 8:**
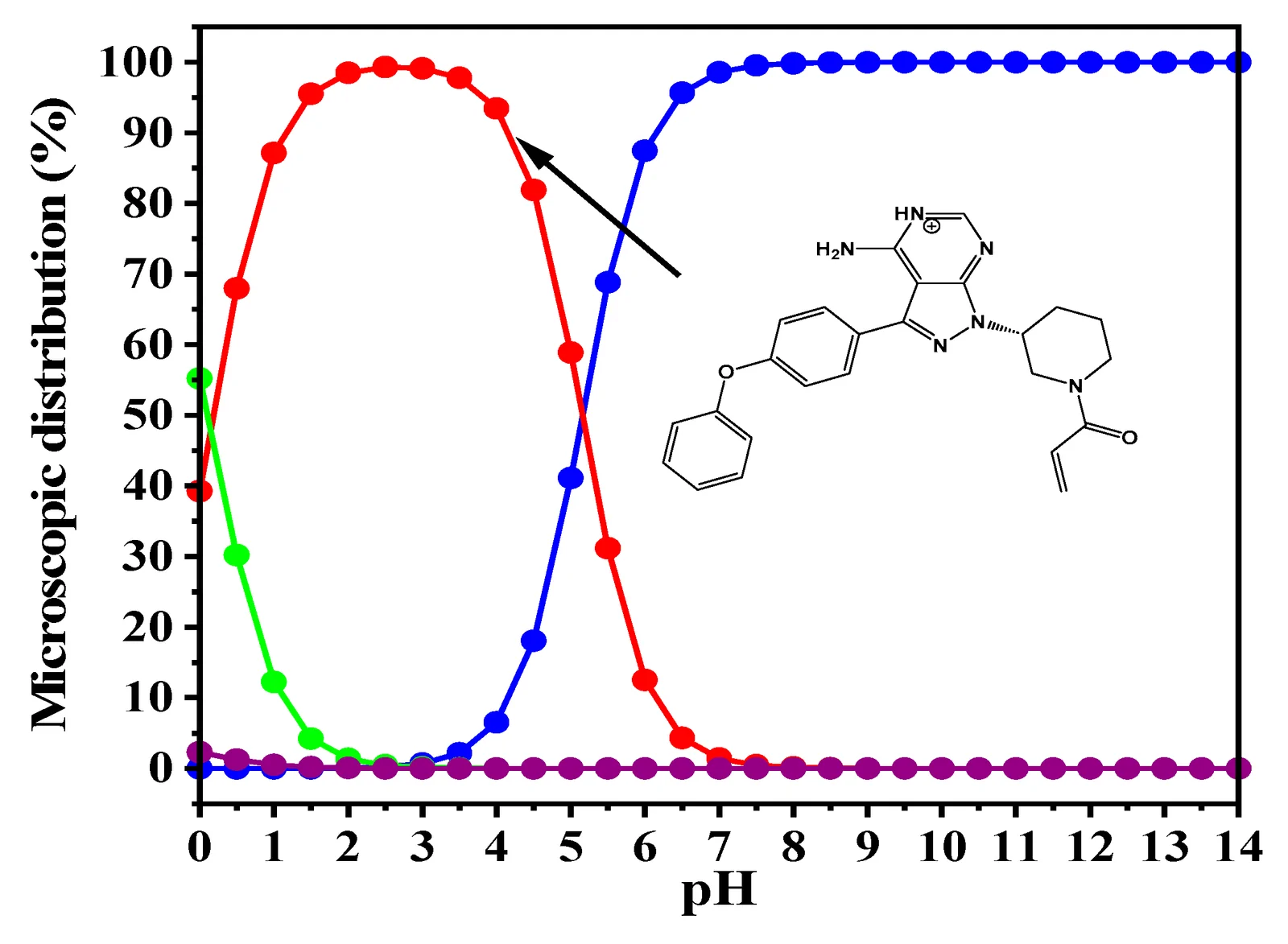
Distribution of the predicted microspecies of EIB as a function of pH, generated using MarvinSketch. The differently colored curves represent the relative distributions of the predicted neutral and protonated microspecies. Under the strongly acidic experimental conditions, the N-protonated form is the predominant microspecies.

The predominant protonation states of the EIB molecule over the pH range of 0–14 were predicted using Marvin Sketch software 25.x. The calculations identified five possible microspecies, including one neutral form and four protonated forms resulting from protonation at the nitrogen atom, the oxygen atom, or both sites. Under strongly acidic conditions, EIB exists almost entirely in its protonated form because hydrochloric acid was used as the corrosive medium in the experimental work (Figure 8). The speciation analysis indicated that the N-protonated species accounted for essentially 100% of the molecular population under these conditions. Therefore, this protonated structure was selected for all subsequent theoretical calculations to ensure consistency with the experimental environment [23].

#### 2.6.2. DFT Calculations

**Figure 9:**
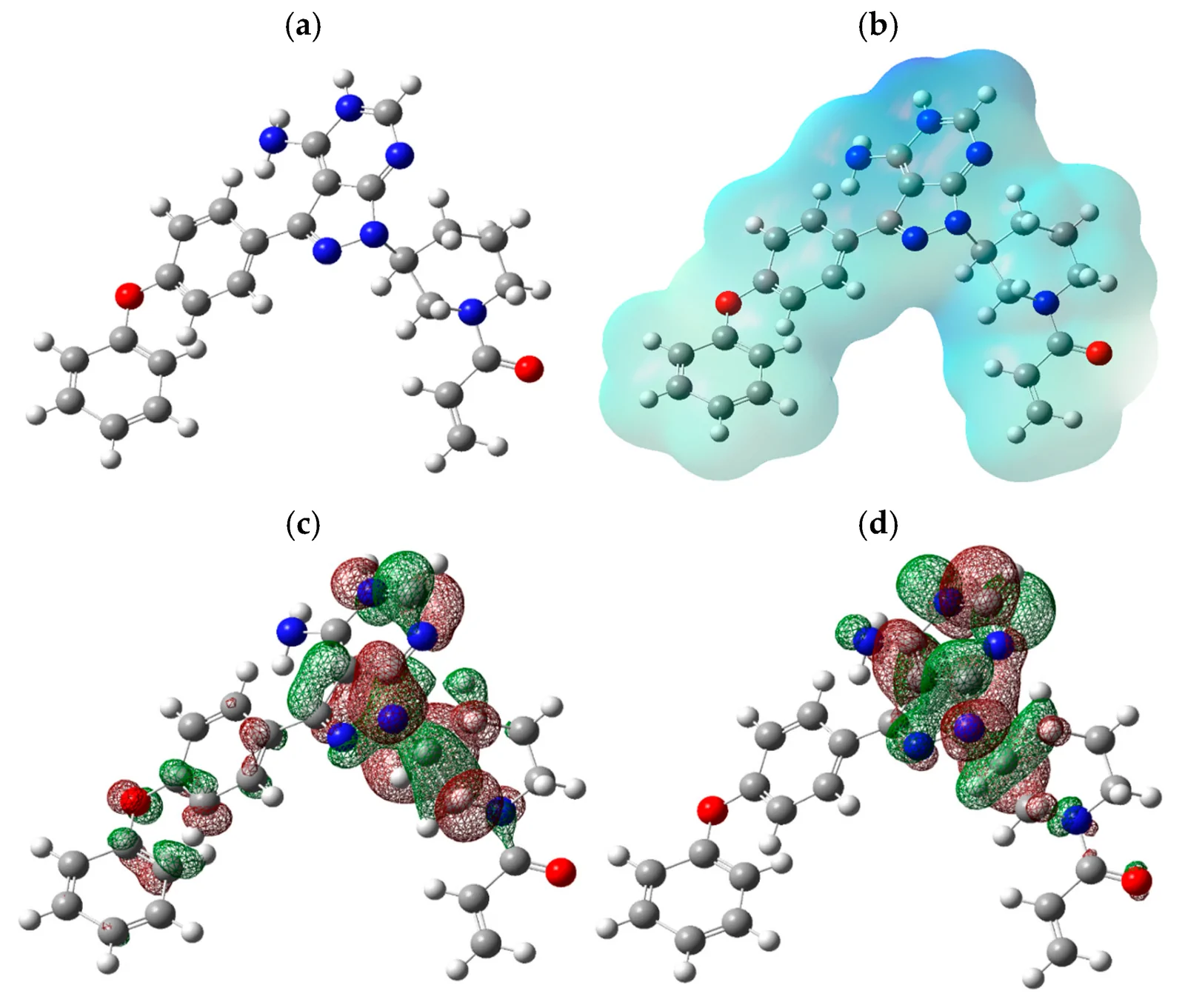
Schematic representation of optimized structures and frontier orbital density distributions and HOMO and LUMO molecular orbitals of the studied molecule EIB: (**a**) Optimized molecule; (**b**) Electrostatic potential; (**c**) HOMO; (**d**) LUMO.

Quantum chemical calculations were performed to investigate the electronic structure of EIB and to gain molecular-level insight into its corrosion inhibition behavior. The molecular geometry was fully optimized using Density Functional Theory (DFT) at the Gaussian computational level, and the electronic properties were analyzed based on Frontier Molecular Orbital (FMO) theory. The optimized molecular structure is shown in Figure 9 while the calculated quantum chemical descriptors, including the HOMO and LUMO energy levels, are summarized in Table 5. The molecular electrostatic potential (MEP) map presented in Figure 9 illustrates the distribution of electron density across the EIB molecule, highlighting the regions most likely to participate in adsorption onto the steel surface. The relatively high HOMO energy indicates that EIB possesses a strong ability to donate electrons to the vacant 3d orbitals of iron atoms, thereby promoting the formation of coordinate bonds with the metal surface [24]. Conversely, the low LUMO energy reflects the molecule’s capacity to accept electrons from the metal, facilitating bidirectional electron transfer during the adsorption process [25]. The HOMO–LUMO energy gap (ΔE) was found to be relatively small, suggesting high molecular reactivity and enhanced adsorption affinity toward the steel surface [26]. Additional global reactivity descriptors further support this behavior. The calculated electronegativity (χ) reflects the tendency of the molecule to attract electrons, whereas the chemical hardness (η) describes its resistance to charge transfer [27]. Furthermore, the electron transfer parameter (ΔN) provides valuable information regarding the direction of electron exchange between the inhibitor and the metal. The fraction of transferred electrons (ΔN) was calculated using Equation (1):

$$∆N=\frac{\chi_{Fe}-\chi_{inh}}{2\left(\eta_{Fe}-\eta_{inh}\right)}$$

where χ_Fe_ = 7.0 eV and η_Fe_ = 0 eV were adopted for bulk iron. Using the calculated electronegativity (χ_inh_ = 6.9299 eV) and hardness (η_inh_ = 0.8274 eV) of EIB, a ΔN value of +0.0424 was obtained. The positive value indicates a predicted tendency for electron transfer from EIB toward the iron surface, supporting donor–acceptor interactions between the inhibitor and Fe.

**Figure 10:**
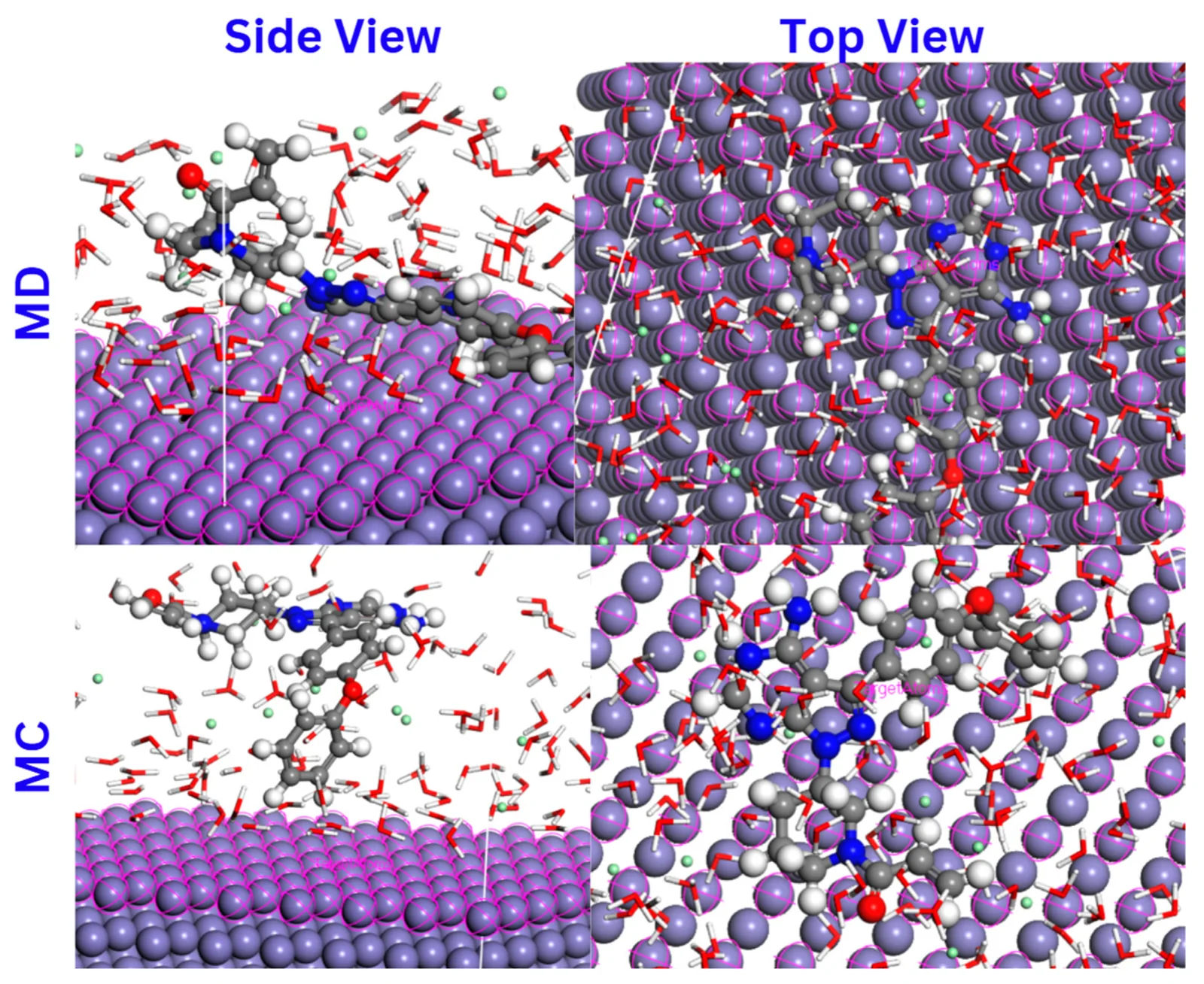
The equilibrium molecular configurations derived from MC and MD simulations for the most favorable modes of adsorption for the (EIB/200 H_2_O/19 H_3_O^+^/20 Cl^−^) solutions on the Fe(110) surface.

The most favorable structure of the EIB inhibitor in Figure 10 is an open planar shape, which may exhibit a very high tendency towards interaction with the steel surface and the generation of active complexes [28]. The distribution of electrons of the HOMO is found around heteroatoms (N, O) and the core of the aromatic ring, displaying electron donation from the EIB inhibitor to d-orbitals of iron by forming chemical bonds. As a counterpart, LUMO electron localization is positioned at the center of the molecule, recognizing its electron-accepting interaction with the steel substrate [29]. HOMO and LUMO regions are the regions of maximum reactivity that function as electrophilic as well as nucleophilic centers. Their general positioning across the EIB structure further activates its absorption ability in terms of donating and accepting an electron to facilitate the formation of an insulating film. The dipole moment (μ) values in Table 5 also indicate the high polarizability and solubility in corrosive acidic environments of EIB molecules [30]. Additionally, the displacement of water molecules from the steel surface is also because of the higher dipole moment of the EIB inhibitor (10.8149 Debye) compared to water (1.84 Debye), which improves its strong interaction with Fe atoms [31].

The interaction of electric charge magnitude with the potential of electric charge to donate can be explored through Natural Bond Orbital (NBO) analysis [32]. The procedure helps us to understand the impact of charge magnitude on charge transfer. Hybridization outcomes and orbital traits using NBO calculations through the B3LYP/6-31G method were given and presented in Table 6. The Table outlines the high-energy donating orbitals of the IEB compound, identifying their type, occupancy, energy, and hybridization. The information shows that terminal heteroatoms and aromatic fragments such as C_14_–C_15_, C_12_–C_13_, LP(2)O_26_, C_10_–C_11_, C_5_–C_6_, C_16_–C_17_, C_7_–C_8_, LP(1)N_22_, and LP(2)O_9_ have lone pairs and π bonds at high energies and are therefore the most probable charge donors.

#### 2.6.3. Molecular Dynamics and Monte Carlo Simulations (MD/MC)

**Figure 11:**
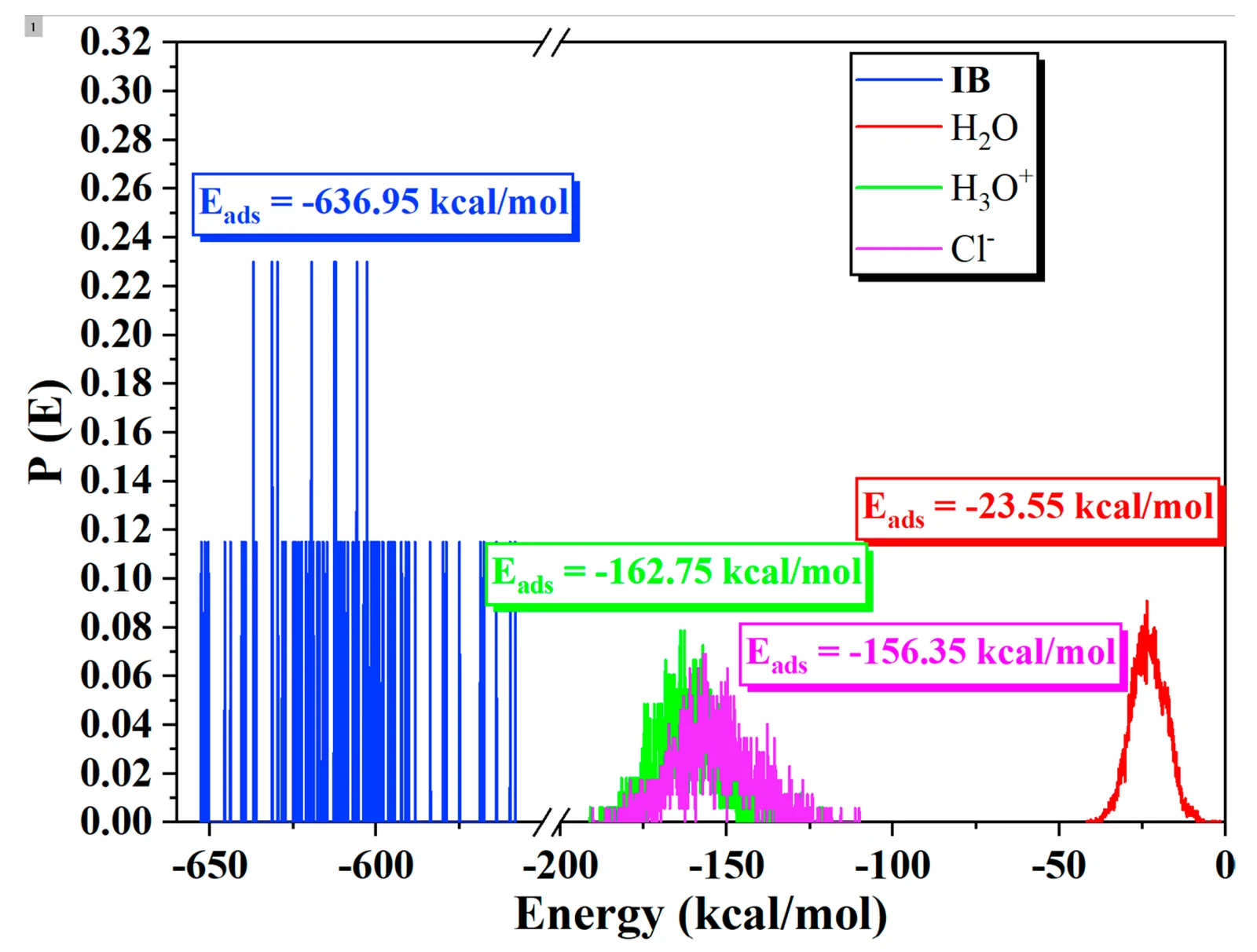
Distribution of the E_ads_ of the (PEOB/300 H_2_O/20 H_3_O^+^/20 Cl^−^) system based on the Monte Carlo simulation.

**Figure 12:**
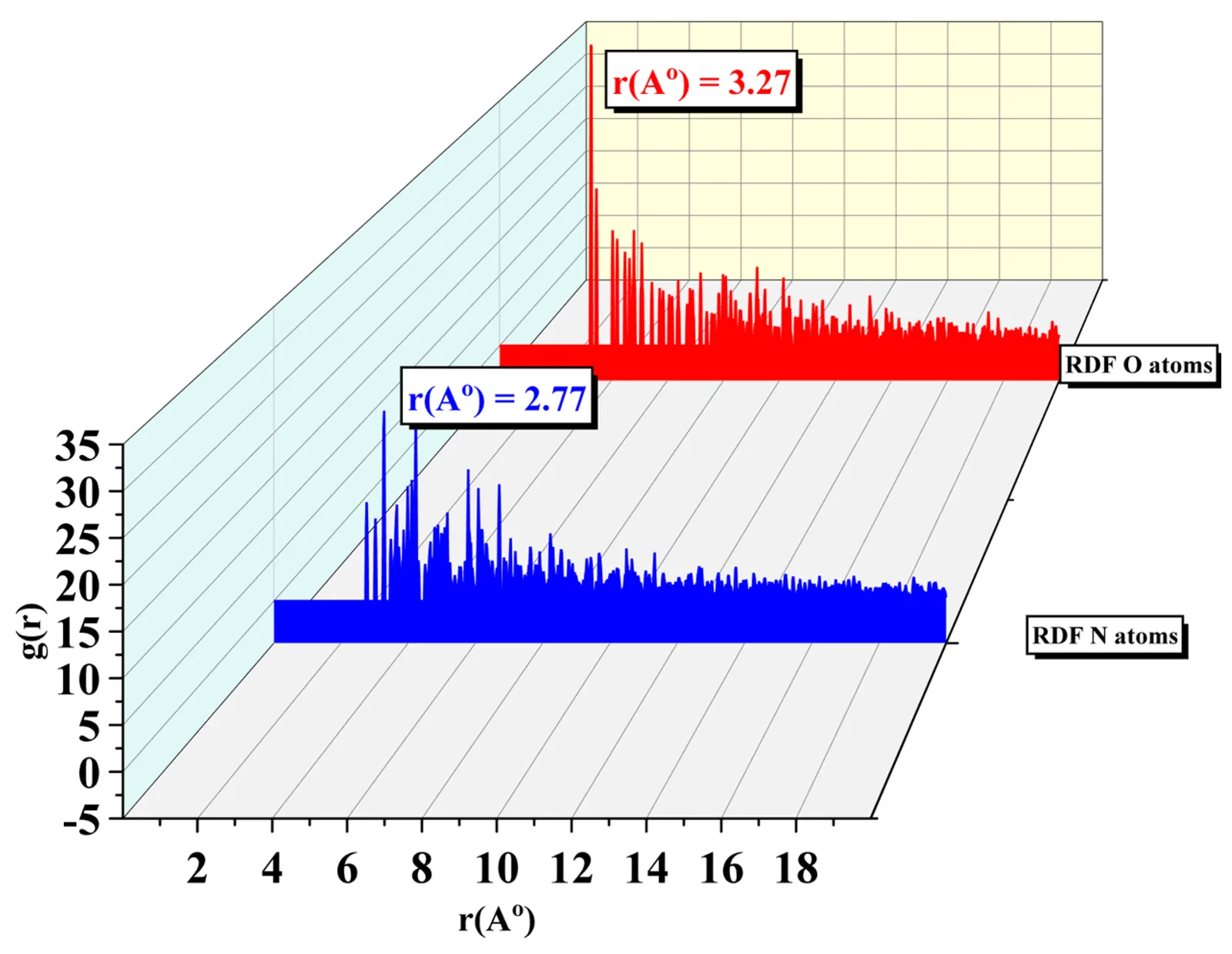
Radial distribution functions for the O and N atoms within the EIB inhibitor/Fe(110) were obtained via molecular dynamics simulations.

Monte Carlo (MC) and molecular dynamics (MD) simulations were carried out in order to study the mechanisms by which EIB molecules suppress corrosion on the iron surface and to look for the minimum energy adsorption sites (E_ads_). The goal was to identify the favored adsorption characteristics of EIB on the Fe(110) surface. Figure 11 displays the adsorption equilibrium geometries of the EIB molecule on iron, along with the respective simulation results. As indicated by Figure 11, it is clear that EIB molecules take a nearly planar conformation when adsorbed on the iron surface, which shows strong interaction. The values of energy of adsorption (E_ads_) derived from Monte Carlo simulations are shown in Figure 12. We find that the EIB molecule has dramatically enhanced adsorption (Eads = −636.95 kcal/mol) compared to other species in the corrosive environment [Water molecules: E_ads_ = −23.55 kcal/mol, Hydronium ions (H_3_O^+^): E_ads_ = −162.75 kcal/mol, Chloride ions (Cl^−^): E_ads_ = −156.35 kcal/mol]. These values suggest that EIB molecules can progressively replace pre-adsorbed H_2_O, H_3_O^+^ and Cl^−^ [33]. The EIB’s strong adsorption of the inhibitor can be ascribed primarily to the formation of Fe-N and Fe-O bonds, while dipole–dipole interaction and electronic charge transfer between the inhibitor and the metal surface are also contributory.

The radial distribution function (RDF) method was employed in order to quantify the iron-bond lengths with the component atoms of the EIB molecule (Figure 12). The method of analysis enabled the identification of the adsorption type from the bond distances. The RDF plot gives the following details: Peaks in the range of 1.0 Å to 3.5 Å indicate chemisorption, where chemical bonds are formed between the metal and the adsorbate. Peaks above 3.5 Å suggest physisorption, as governed by the weaker van der Waals forces [34]. As indicated in Figure 9, EIB molecules contain heteroatoms positioned near the iron surface, indicating strong adsorption. This interaction corroborates the efficiency of EIB as a corrosion inhibitor by forming a protective film on the steel surface that is stable. The results highlight the role of chemical bonding and electronic effects in enhancing the corrosion resistance of metals.

## 3. Materials and Methods

### 3.1. Materials

The P110 carbon steel (CS) with weight percentages of 0.25% C, 1.10% Mn, 0.03% P, 0.025 S, 0.40 Si, and Fe was used in this study. The carbon steel (CS) samples used had dimensions of 5.0 cm × 3.0 cm × 0.25 cm. Prior to testing, the surfaces were polished sequentially with 400-, 600-, and 800-grit emery papers to ensure smoothness. After polishing, the samples were rinsed with a water–acetone mixture to remove any residues and then thoroughly dried. Drugs with a purity of 99%, manufactured by IMBRUVICA (South San Francisco, CA, USA), were expired for about one year.

### 3.2. Methods

#### 3.2.1. Structural Analysis and Surface Studies

To analyze the inhibitor’s functional groups, FTIR was employed using an instrument covering a wavenumber range of 400–4000 cm^−1^. The surface morphology was examined using scanning electron microscopy (SEM) with a JEM-2100F instrument, JOEL LTD, Akishima, Japan.

#### 3.2.2. Electrochemical Tests

Electrochemical measurements were carried out on carbon steel (CS) coupons with an exposed surface area of 1 cm^2^. A standard three-electrode cell configuration was used, where the CS coupon served as the working electrode, a saturated calomel electrode (SCE) functioned as the reference electrode, and a platinum electrode acted as the counter electrode. All electrochemical tests were conducted using a SP-200 Biologic potentiostat (Seyssinet-Pariset, France). For electrochemical impedance spectroscopy (EIS), the measurements were performed over a frequency range of 10 kHz to 100 mHz with a sinusoidal perturbation amplitude of 10 mV. Potentiodynamic polarization (PDP) studies were carried out at a scan rate of 1 mV/s across a potential window of ±250 mV around the open circuit potential (OCP) for 30 min.

#### 3.2.3. Mass Loss Method

Mass loss measurements were carried out in accordance with ASTM G31-21, “Standard Guide for Laboratory Immersion Corrosion Testing of Metals [35]. Pre-weighed P110 carbon steel specimens were polished using successive grades of silicon carbide abrasive papers, degreased with acetone, rinsed thoroughly with distilled water, and dried before immersion. The specimens were immersed in 1.0 M HCl solution in the absence and presence of different concentrations of EIB (50–1000 mg L^−1^) for 6 h at the desired temperature under stagnant conditions. After immersion, the specimens were removed, rinsed with distilled water, cleaned to remove corrosion products according to the ASTM G1 cleaning procedure [36], washed with acetone, dried, and reweighed. The corrosion rate was determined from the measured mass loss and expressed as mg cm^−2^ h^−1^. The inhibition efficiency (IE%) was calculated using the following equation:

(3) $$EI\%=\frac{W_{0}-W_{i}}{W_{0}}\times 100$$

where W_0_ and W_i_ are the mass loss values of P110 carbon steel in the absence and presence of EIB, respectively.

#### 3.2.4. Computational Investigations

The molecular structure of ibrutinib (IB) was optimized using Density Functional Theory (DFT) at the B3LYP/6-31G level implemented in the Gaussian 16 package [37]. Geometry optimization confirmed that the molecule reached its minimum-energy configuration, providing a reliable basis for subsequent electronic structure and adsorption analyses. To further elucidate the electronic characteristics of IB, Natural Bond Orbital (NBO) analysis was performed to evaluate its bonding features, charge distribution, hybridization, orbital interactions, and the electronic properties of potential adsorption centers [38].

The adsorption behavior of IB on the Fe(110) surface was subsequently investigated using both molecular dynamics (MD) and Monte Carlo (MC) simulations. MD simulations were carried out with the Forcite module, whereas MC calculations were performed using the Adsorption Locator module [32]. The simulation cell consisted of an Fe(110) slab in contact with a corrosive solution containing 200 water molecules, 20 hydronium ions (H_3_O^+^), and 20 chloride ions (Cl^−^). The Fe(110) surface was selected because of its high stability and its widespread use as a representative model for iron corrosion studies.

Molecular dynamics simulations were conducted at 298 K under the canonical (NVT) ensemble, maintaining constant temperature and volume through a Nosé thermostat. Each simulation was run for 50 ps with a time step of 1 fs, allowing sufficient sampling of the molecular adsorption process and interfacial interactions. The COMPASS II force field was employed for all energy calculations owing to its proven accuracy in describing intermolecular interactions, adsorption behavior, and molecular stability at metal/solution interfaces [39]. This multiscale computational approach provided detailed insights into the adsorption configuration of IB, the strength of its interaction with the Fe(110) surface, and its ability to form a stable protective film that effectively suppresses corrosion.

## 4. Conclusions

The corrosion inhibition performance of expired ibrutinib (EIB) toward P110 carbon steel in 1.0 M HCl was systematically evaluated using electrochemical techniques, gravimetric analysis, surface characterization, and theoretical calculations. The results consistently demonstrated that EIB effectively suppresses steel corrosion by adsorbing onto the metal surface and forming a compact protective film. Electrochemical measurements showed that the charge-transfer resistance increased while the corrosion current density markedly decreased with increasing inhibitor concentration, indicating improved corrosion resistance. The highest inhibition efficiencies reached 88.7%, 96.8%, and 93.3% according to EIS, potentiodynamic polarization, and mass loss measurements, respectively, at 1000 mg L^−1^ EIB. The calculated thermodynamic parameters suggested a spontaneous adsorption process involving both physical and chemical interactions. SEM test further confirmed the formation of a smoother and more homogeneous steel surface in the presence of EIB compared with the severely damaged uninhibited specimen. DFT, NBO, Monte Carlo, molecular dynamics, and RDF analyses revealed that the heteroatoms and π-electron system of EIB strongly interact with the Fe(110) surface through Fe–N and Fe–O interactions, providing molecular-level evidence for the experimentally observed inhibition behavior. Overall, the excellent agreement between the experimental and computational findings demonstrates that expired ibrutinib is a highly effective and sustainable corrosion inhibitor for P110 carbon steel in acidic media, while also offering an environmentally responsible approach for the beneficial reuse of expired pharmaceutical products.

## Figures and Tables

**Table 1:** The obtained electrochemical parameters from data modeling of the EIS results.

Conc. (mg L^−1^)	Rs (Ohm·cm^2^)	CPE_dl_,Y_0_ (mS·s^n^/cm^2^)	*n*	Rct (Ohm·cm^2^)	Cdl (µF/cm^2^)	IE (%)
Blank	0.298	4.76	0.82	18.3	1124	-
250	0.187	1.32	0.88	57.8	425	67.5
500	0.433	1.04	0.91	75.1	485	75.6
750	0.411	0.75	0.91	99.2	337	81.6
1000	0.486	0.64	0.94	161.3	382	88.7

**Table 2:** Electrochemical parameters obtained from PDP measurements for P110 carbon steel in 1.0 M HCl containing different concentrations of EIB.

EIB Dosage (mg L^−1^)	β_a_ (V/dec)	−β_c_ (V/dec)	E_corr_ vs. SCE (V)	i_corr_ (µA cm^−2^)	Rp (kΩ cm^2^)	IE%
Blank	0.116	0.174	−0.480	1.954	15.5	--
250	0.115	0.176	−0.479	0.609	49.6	68.9
500	0.116	0.175	−0.481	0.536	56.5	72.9
750	0.115	0.177	−0.478	0.297	101.9	84.8
1000	0.117	0.175	−0.482	0.062	491.1	96.8

**Table 3:** Mass loss and inhibition efficiency of EIB for P110 CS in 1.0 M HCl.

Conc. (mg L^−1^)	ML (mg cm^−2^ h^−1^)	EI%
Blank	0.2003	--
50	0.1160	42.1
100	0.0923	53.9
250	0.0724	63.9
500	0.0516	74.2
750	0.0352	82.4
1000	0.0134	93.3

**Table 4:** Effect of temperature on corrosion rate in the presence and absence of EIB.

T, K	Conc. (mg L^−1^)	ML (mg)	CR (mg cm^−2^ h^−1^)
298	Blank	0.2003	0.4922
308		0.2772	0.6812
318		0.3361	0.8259
328		0.3976	0.9770
298	1000	0.0134	0.0329
308		0.0286	0.0703
318		0.0458	0.1125
328		0.0702	0.1725

**Table 5:** Reactivity indices of EIB molecule, calculated by B3LYP level.

Calculated Indices	EIB
E_LUMO_	−6.1025
E_HOMO_	−7.7573
ΔE (E_LUMO_ − E_HOMO_)	1.6548
∆E back donation	−0.2068
Dipole moment	10.8149
Total negative charge	−6.5622
Chemical potential (μ)	−6.9299
Chemical hardness (η)	0.8274
Chemical softness (σ)	1.2086
Electronegativity (χ)	6.9299
Global electrophilicity index (ω)	29.0212
Electron-donating power (ω−)	32.5895
Electron-receiving power (ω+)	25.6596
ΔN	+0.0424

**Table 6:** Hybridization data for EIB inhibitor from B3LYP/6-31g calculations.

Structure	Type	Occ.	Energy	NBOs	*p*% Atom 1	*p*% Atom 2
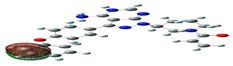	$C_{14}-C_{15}$	1.80785	−0.33637	0.7018 P + 0.7123 P	100.00	100.00
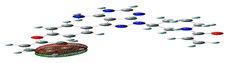	$C_{12}-C_{13}$	1.81820	−0.33832	0.7149 SP + 0.6992 SP	99.93	99.95
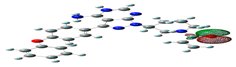	$LP(2)O_{26}$	1.84663	−0.35022	SP	99.58	----
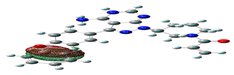	$C_{10}-C_{11}$	1.76812	−0.35513	0.6978 SP + 0.7163 SP	99.99	99.32
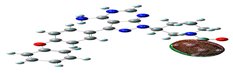	$C_{24}-C_{25}$	1.90755	−0.36650	0.7457 SP + 0.663 SP	99.98	99.98
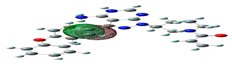	$C_{5}-C_{6}$	1.76219	−0.37240	0.7413 P + 0.6711 SP	100.00	99.99
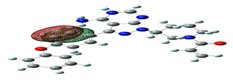	$C_{16}-C_{17}$	1.82910	−0.37504	0.6923 SP + 0.7216 SP	99.97	99.97
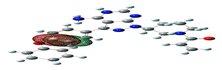	$C_{7}-C_{8}$	1.74460	−0.37537	0.7217 SP + 0.6923 SP	99.91	99.99
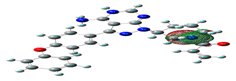	$LP(1)N_{22}$	1.77454	−0.41351	SP	83.81	------
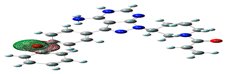	$LP(2)O_{9}$	1.79988	−0.41595	SP	98.20	------

## Data Availability

The raw data supporting the conclusions of this article will be made available by the authors on request.
